# Hypertension and High Blood Pressure Are Associated With Dementia Among Chinese Dwelling Elderly: The Shanghai Aging Study

**DOI:** 10.3389/fneur.2018.00664

**Published:** 2018-09-04

**Authors:** Xiaoniu Liang, Ying Shan, Ding Ding, Qianhua Zhao, Qihao Guo, Li Zheng, Wei Deng, Jianfeng Luo, Lap A. Tse, Zhen Hong

**Affiliations:** ^1^Institute of Neurology, Huashan Hospital, Fudan University, Shanghai, China; ^2^National Clinical Research Center for Aging and Medicine, Huashan Hospital, Fudan University, Shanghai, China; ^3^Department of Cardiology, Huashan Hospital, Fudan University, Shanghai, China; ^4^Department of Biostatistics, School of Public Health, Fudan University, Shanghai, China; ^5^Key Laboratory of Public Health Safety of Ministry of Education, Shanghai, China; ^6^JC School of Public Health and Primary Care, The Chinese University of Hong Kong, Hong Kong, Hong Kong

**Keywords:** cognitive function, dementia, mild cognitive impairment, hypertension, blood pressure, community-based study

## Abstract

**Background:** To explore the association between blood pressure and cognition in older participants in the Shanghai Aging Study.

**Methods:** Data were drawn from 3,327 participants at the baseline of Shanghai Aging Study. History of hypertension was inquired and confirmed from participants' medical records. Systolic blood pressure (SBP) and diastolic blood pressure (DBP) were measured by research nurses in the early morning. Participants were diagnosed with “cognitive normal,” “mild cognitive impairment (MCI),” or “dementia” by neurologists using DSM-IV and Petersen criteria. Multivariate logistic regression was used to evaluate the association between history of hypertension, duration of hypertension, SBP, DBP, or classification of blood pressure and cognitive function. Generalized linear model was used to assess the relation between duration of hypertension, SBP, or DBP and Mini Mental State Examination (MMSE).

**Results:** A significantly higher proportion of hypertension [78 (76.5%)] was found in participants with dementia than in those with MCI [347 (59.3%)] and cognitive normal [1,350 (51.1%)] (*P* < 0.0001). Participants with dementia had significantly higher SBP [157.6 (26.1) mmHg] than those with MCI [149.0 (23.7) mmHg] and cognitive normal [143.7 (22.6) mmHg] (*P* < 0.0001). After adjusting for sex, age, education, living alone, body mass index, anxiety, depression, heart disease, diabetes, and stroke, the likelihood of having dementia was positively associated with history of hypertension (*OR* = 2.10; 95% CI: 1.22, 3.61), duration of hypertension (*OR* = 1.02 per increment year; 95% CI: 1.01, 1.04), higher SBP (*OR* = 1.14 per increment of 10 mmHg; 95% CI: 1.04, 1.25), higher DBP (*OR* = 1.22 per increment of 10 mmHg; 95% CI: 1.02, 1.45), moderate hypertension (*OR* = 2.09; 95% CI: 1.10, 3.99), or severe hypertension (*OR* = 2.45; 95% CI: 1.20, 4.99). The MMSE score was inversely correlated to duration of hypertension (β = −0.0088 per increment year; 95% CI: −0.0158, −0.0018, *P* = 0.0132), SBP (β = −0.0655 per increment of 10 mmHg; 95% CI: −0.1022, −0.0288, *P* = 0.0005), and DBP (β = −0.1230 per increment of 10 mmHg; 95% CI: −0.1915, −0.0545, *P* = 0.0004).

**Conclusion:** Our results suggest that hypertension and high blood pressure may be potential risk factors for dementia. Blood pressure management for the elderly may be important for maintaining cognitive vitality.

## Introduction

Cognitive impairment has a great impact on disability and mortality among the elderly, while it also reduces the quality of life for both patients and their caregivers. Approximately, there have been 24.3 million prevalent dementia cases, with 4.6 million new cases worldwide every year (1). China had 9.2 million cases of dementia in 2010 (2), and the case number will rise to the world's top by 2025 (1).

Hypertension is a highly prevalent condition, occurring in one-third of the world's adults and in two-thirds of adults over 65 years of age (3, 4). Both hypertension and dementia are age-related comorbidities which may induce considerable disabilities (1, 5–7). Some epidemiological studies showed that hypertension is an important risk factor of dementia (8, 9), which was evident from the positive relationship between blood pressure at midlife and the subsequently higher risk of cognitive impairment or dementia late in life (10–12); however, some other studies provided contradictory evidence that low blood pressure was a risk factor for dementia and cognitive decline (13–15). Until now, the relationship between hypertension and dementia or cognitive decline has been inconsistent, and mixed findings have been reported from cross-sectional and longitudinal studies (6, 16–18).

Epidemiological dementia research in China is still far behind developed countries. Only two epidemiological studies examined the relation between hypertension and cognitive impairment in the elderly in China, but it was inconclusive because of small sample size and simple neuropsychological assessments (19, 20). The Shanghai Aging Study intended to identify the prevalence and the incidence of dementia and mild cognitive impairment (MCI) among a cohort of old adults in an urban community in Shanghai, China (21). We, therefore, intend to explore the association between blood pressure and cognition in this cohort by analyzing the baseline data of the Shanghai Aging Study.

## Materials and methods

### Recruitment of participants

From January 2010 to December 2012, the Shanghai Aging Study recruited 3,836 permanent residents aged ≥50 years in the Jingansi community, Shanghai. Participants were excluded if they were (1) residing in nursing homes or other institutions; (2) suffering from severe schizophrenia or mental retardation, based on the data abstracted from their medical record or diagnosed by neurologists; or (3) suffering from severe vision, hearing, or verbal impairment and could not participate in the neuropsychological evaluation. Detailed process of the participant recruitment has been published elsewhere (21). In this study, we used the dataset of 3,327 participants aged 60–85 years.

This study was approved by the Medical Ethics Committee of Huashan Hospital, Fudan University, Shanghai, China. All the participants and/or their legal guardians provided their written informed consent to participate in the study.

### Demographic characteristics and medical history

Participants were interviewed face-to-face by neurologists and research nurses to collect information on their demographic characteristics, including age, sex, and education. The participants' weight and height were measured and used to calculate the body mass index (BMI: the weight in kilograms divided by the square of the height in meters). We also obtained lifestyle factors, such as living alone, cigarette smoking, and alcohol drinking. Their history of chronic diseases, such as diabetes, stroke, and heart disease (including coronary artery disease and arrhythmia), was inquired and confirmed from their medical records.

### Blood pressure measurement

From 7:30 to 8:00 a.m., after at least 5 min of rest in a seated position blood pressure was measured twice, with a standard sized cuff placed on the right arm at heart level of the seated subject, by trained research nurses using a validated and calibrated digital electronic tensiometer (M4; OMRON Corp., Kyoto, Japan) (22). We recorded the blood pressure as the mean of two measurements of systolic blood pressure (SBP) and diastolic blood pressure (DBP).

### Hypertension and classification of blood pressure

Hypertension was defined as the reported physician-diagnosed hypertension, with or without treatment. Duration of hypertension was also inquired and confirmed from the participants' medical records (19, 23). According to the 2010 Chinese guidelines for the management of hypertension, blood pressure was classified into four categories: severe hypertension: SBP ≥180 mmHg or DBP ≥110 mmHg; moderate hypertension: SBP of 160–179 mmHg or DBP of 100–109 mmHg; mild hypertension: SBP of 140–159 mmHg or DBP of 90–99 mmHg; and normal blood pressure: SBP < 140 mmHg and DBP < 90 mmHg (23).

### Neuropsychological assessments

Cognitive function of each participant was assessed by a neuropsychological test battery, which covers domains of global cognition, executive function, spatial construction function, memory, attention, and language. The battery contained (1) Mini Mental State Examination (MMSE); (2) Conflicting Instructions Task (Go/No Go Task); (3) Stick Test; (4) Modified Common Objects Sorting Test; (5) Auditory Verbal Learning Test; (6) Modified Fuld Object Memory Evaluation; (7) Trail-making test A&B; and (8) RMB (Chinese currency) test. Neuropsychological tests were administered by study psychometrists according to the different education levels of each participant. Test 1, 2, 3, 4, 5, and 7 in the battery were used in participants with education ≥6 years; test 1, 2, 3, 4, 6, and 8 were used in those with education < 6 years. All tests were conducted in Chinese within 90 min. A pilot validation study was conducted for each cognitive measure using corrections for gender, age, and years of education in a community of healthy elderly people, and the normative data and the detailed description of these tests were reported elsewhere (24, 25).

### Neurological exams

Neurologists examined each participant for their reflexes and motor responses. They also administered the Center for Epidemiologic Studies Depression Scale (CES-D) (26) and the Zung Self-Rating Anxiety Scale (SAS) (27) to evaluate whether each participant met the criteria of having a major depression (CES-D ≥ 16) or anxiety (SAS > 44) episode within the past week. Neurologists also administered the Clinical Dementia Rating (CDR) (28, 29) and Activities of Daily Living (ADL) (30) scale to obtain information on cognitive complaints and activities of daily living.

### Consensus diagnoses

After each clinical assessment, two study neurologists, one neuropsychologist, and one neuroepidemiologist reviewed the functional, medical, neurological, psychiatric, and neuropsychological data and reached a consensus regarding the presence or absence of dementia using DSM-IV criteria (31). Only those who were not diagnosed with dementia were considered for the diagnosis of MCI, which was defined according to Petersen's criteria (32). Diagnostic procedures were reported elsewhere (21). Based on the consensus diagnoses, the subjects were classified into three groups with dementia, MCI, and cognitive normal.

### APOE genotype assessment

DNA was extracted from blood or saliva collected from the study participants. *Apolipoprotein E (APOE)* genotyping was conducted by the TaqmanSNP method (33). The presence of at least one ε*4 allele* was defined as being *APOE*-ε*4* positive.

### Statistical analyses

Continuous variables were expressed as the mean ± standard deviation (SD) or median (25%, 75%), and categorical variables were expressed as frequencies (%). The analysis of variance (ANOVA) and Kruskal-Wallis test were used to compare the continuous variables; the Cochran-Mantel-Haenszel Chi-squared test was used to compare the categorical variables. Multivariate logistic regression model was used to detect the association between history of hypertension, duration of hypertension, SBP, DBP, or blood pressure categories and different clinical cognitive diagnoses, which were adjusted for confounders. Measurement of the association was presented as odds ratio (OR) and 95% confidence interval (CI). Generalized linear model was used to evaluate the relation between duration of hypertension, SBP, or DBP and MMSE, which were adjusted for confounders. Systolic and diastolic blood pressures were regarded as continuous variables and were expressed in units of 10 mmHg (original blood pressure value divided by 10) in the multivariate logistic regression model and in the generalized linear model.

All the *P*-values and 95% CIs were estimated in two-tailed tests. Differences were considered to be statistically significant at *P* < 0.05. The data analysis was conducted using SAS 9.4 (SAS Institute Inc., Cary, NC, USA).

## Results

### Demographic, lifestyles, and medical history of the participants

Among the 3,327 participants, 1,519 (45.7%) were males. The mean age of all the participants was 70.1 (SD 7.2) years, and the mean year of education was 11.7 (SD 4.2) years. Five hundred and eighty-five (17.6%) participants were diagnosed as MCI, while 102 (3.1%) were diagnosed as dementia. Age, education, MMSE score, alcohol drinking, *APOE*-ε*4 allele*, history of heart disease, diabetes, stroke, anxiety, and depression were found to be significantly different across groups with different diagnosis of cognition (Table 1).

**Table 1:** Demographic, lifestyles, and medical history of the participants with cognitive normal, MCI, and dementia.

	**All (*n* = 3,327)**	**Cognitive normal (*n* = 2,640)**	**MCI (*n* = 585)**	**Dementia (*n* = 102)**	*P*-Value^*^
Age, years, mean ± SD	70.1 ± 7.2	69.2 ± 6.9	73.1 ± 7.3	77.4 ± 6.0	< 0.0001
Education, years, mean ± SD	11.7 ± 4.2	12.2 ± 3.8	9.9 ± 4.8	8.0 ± 6.0	< 0.0001
MMSE, scores, mean ± SD	27.9 ± 2.8	28.6 ± 1.7	26.5 ± 2.9	17.5 ± 4.8	< 0.0001
BMI, kg/m^2^, mean ± SD	24.4 ± 3.6	24.3 ± 3.4	24.6 ± 4.2	23.9 ± 3.9	0.1635
Sex, male*s, n* (%)	1519 (45.7)	1216 (46.1)	261 (44.6)	42 (41.2)	0.2815
Cigarette smoking, *n* (%)	359 (10.8)	280 (10.6)	71 (12.2)	8 (7.8)	0.8389
Alcohol drinking, *n* (%)	261 (7.9)	219 (8.4)	40 (6.9)	2 (2.0)	0.0193
Anxiety, *n* (%)	74 (2.2)	51 (1.9)	18 (3.1)	5 (5.0)	0.0118
Depression, *n* (%)	564 (17.0)	408 (15.5)	126 (21.7)	30 (29.4)	< 0.0001
Heart disease, *n* (%)	407 (12.3)	289 (11.0)	97 (16.6)	21 (20.6)	< 0.0001
Diabetes, *n* (%)	459 (13.8)	338 (12.8)	104 (17.8)	17 (16.7)	0.0028
Stroke, *n* (%)	413 (12.4)	272 (10.3)	102 (17.5)	39 (38.2)	< 0.0001
Living alone, *n* (%)	287 (8.6)	219 (8.3)	63 (10.8)	5 (4.9)	0.5353
*APOE*-ε4 *allele* positive, *n* (%)	564 (18.1)	437 (17.5)	106 (19.9)	21 (25.0)	0.0403

*SD, standard deviation; MCI, mild cognitive impairment; MMSE, Mini-Mental State Examination; BMI, body mass index; APOE, Apolipoprotein*.

* *P-Value is for the comparison among three groups of participants with cognitive normal, MCI, and dementia*.

### Hypertension and blood pressure of groups with different cognition

One thousand seven hundred and seventy-five (53.4%) participants were suffering from hypertension. Participants with dementia had a significantly higher proportion of having hypertension (76.5%) than those with MCI (59.3%) and cognitive normal (51.1%) (*P* < 0.0001). Participants with dementia had significantly longer duration of hypertension (median 10 years) than those with MCI (median 4 years) and cognitive normal (median 1 year) (*P* < 0.0001). Participants with dementia had significantly higher SBP [157.6 mmHg (SD 26.1)] than those with MCI [149.0 mmHg (SD 23.7)] and cognitive normal [143.7 mmHg (SD 22.6)] (*P* < 0.0001). The distribution of blood pressure category was significantly different across the three groups (*P* < 0.001). More moderate and severe hypertension (47.0%) was seen in participants with dementia than those with MCI (33.4%) and cognitive normal (23.5%) (*P* < 0.001; Table 2).

**Table 2:** Hypertension and blood pressure of the participants with cognitive normal, MCI, and dementia.

	**All (*n* = 3,327)**	**Cognitive normal (*n* = 2,640)**	**MCI (*n* = 585)**	**Dementia (*n* = 102)**	*P*-Value^*^
History of hypertension, *n* (%)	1775 (53.4)	1350 (51.1)	347 (59.3)	78 (76.5)	< 0.0001
Duration of hypertension, year, median (25%, 75%)	2 (0, 13)	1 (0, 11)	4 (0, 17)	10 (1, 21.5)	< 0.0001
SBP, mmHg, mean ± SD	145.0 ± 23.1	143.7 ± 22.6	149.0 ± 23.7	157.6 ± 26.1	< 0.0001
DBP, mmHg, mean ± SD	77.3 ± 11.7	77.5 ± 11.6	76.3 ± 12.1	78.4 ± 14.0	0.0735
Classification of BP, *n* (%)					< 0.0001
Normal	1390 (41.8)	1151 (43.6)	212 (36.2)	27 (26.5)	
Mild	1072 (32.2)	867 (32.9)	178 (30.4)	27 (26.5)	
Moderate	608 (18.3)	447 (16.9)	135 (23.1)	26 (25.5)	
Severe	257 (7.7)	175 (6.6)	60 (10.3)	22 (21.5)	

*BP, blood pressure; SBP, systolic blood pressure; DBP, diastolic blood pressure; MCI, mild cognitive impairment; SD, standard deviation*.

* *P-Value is for the comparison among three groups of participants with cognitive normal, MCI, and dementia*.

### Association between blood pressure and MMSE

After adjusting for age, sex, education, BMI, living alone, anxiety, depression, heart disease, diabetes, and stroke in the generalized linear model, the decline in MMSE score was significantly correlated with duration of hypertension (β = −0.0088 per increment year; 95% CI: −0.0158, −0.0018; *P* = 0.0132), SBP (β = −0.0655 per increment of 10 mmHg: 95% CI: −0.1022, −0.0288; *P* = 0.0005), and DBP (β = −0.1230 per increment of 10 mmHg; 95% CI: −0.1915, −0.0545; *P* = 0.0004; Table 3).

**Table 3:** Adjusted association between blood pressure and MMSE.

**Blood pressure**	**β**	**95% CI**	**Wald Chi-square**	***P*-Value**
Duration of hypertension (per increment year)	−0.0088	(−0.0158, −0.0018)	6.14	0.0132
SBP (per increment of 10 mmHg)	−0.0655	(−0.1022, −0.0288)	12.22	0.0005
DBP (per increment of 10 mmHg)	−0.1230	(−0.1915, −0.0545)	12.38	0.0004

*MMSE, Mini-Mental State examination; SBP, systolic blood pressure; DBP, diastolic blood pressure; β, beta coefficient; CI, confidence interval*.

*Generalized linear model adjusted for sex, age, education, body mass index, living alone, anxiety, depression, heart disease, diabetes, and stroke*.

### Association between hypertension, blood pressure, and cognition

After adjusting for age, sex, education, BMI, living alone, anxiety, depression, heart disease, diabetes, and stroke, the likelihood of having dementia was positively associated with history of hypertension (*OR* = 2.10; 95% CI: 1.22, 3.61), duration of hypertension (*OR* = 1.02 per increment year; 95% CI: 1.01,1.04), higher SBP (*OR* = 1.14 per increment of 10 mmHg; 95% CI: 1.04, 1.25), higher DBP (*OR* = 1.22 per increment of 10 mmHg; 95% CI: 1.02, 1.45), moderate hypertension (*OR* = 2.09; 95% CI: 1.10, 3.99), or severe hypertension (*OR* = 2.45; 95% CI: 1.20, 4.99), but it was not associated with mild hypertension (*OR* = 1.31; 95% CI: 0.70, 2.45). There was no significant association between MCI and hypertension or blood pressure (Table 4).

**Table 4:** Adjusted odds ratios for hypertension and blood pressure among participants with dementia vs. cognitive normal, and with MCI vs. cognitive normal [OR (95% CI)].

	**Model 1**	**Model 2**
**DEMENTIA VS. COGNITIVE NORMAL**		
History of hypertension	3.11 (1.95, 4.94)	2.10 (1.22, 3.61)
Duration of hypertension (per increment year)	1.04 (1.02, 1.05)	1.02 (1.01, 1.04)
SBP(per increment of 10 mmHg)	1.27 (1.18, 1.38)	1.14 (1.04, 1.25)
DBP(per increment of 10 mmHg)	1.06 (0.90, 1.25)	1.22 (1.02, 1.45)
Classification of BP		
Normal	1 (reference)	1 (reference)
Mild	1.33 (0.77, 2.28)	1.31 (0.70, 2.45)
Moderate	2.48 (1.43, 4.30)	2.09 (1.10, 3.99)
Severe	5.36 (2.99, 9.62)	2.45 (1.20, 4.99)
**MCI VS. COGNITIVE NORMAL**		
History of hypertension	1.39 (1.16, 1.67)	1.09 (0.89, 1.34)
Duration of hypertension (per increment year)	1.02 (1.01, 1.02)	1.01 (0.99, 1.01)
SBP(per increment of 10 mmHg)	1.10 (1.06, 1.15)	1.03 (0.99, 1.08)
DBP(per increment of 10 mmHg)	0.91 (0.84, 0.98)	0.98 (0.90, 1.06)
Classification of BP		
Normal	1 (reference)	1 (reference)
Mild	1.12 (0.90, 1.39)	0.99 (0.79, 1.26)
Moderate	1.64 (1.29, 2.09)	1.28 (0.98, 1.67)
Severe	1.86 (1.34, 2.58)	1.11 (0.77, 1.60)

*BP, blood pressure; SBP, systolic blood pressure; DBP, diastolic blood pressure; MCI, mild cognitive impairment; OR, odds ratio; CI, confidence interval*.

*Univariate logistic regression model 1 didn't adjust any confounder*.

*Multivariate logistic regression model 2 adjusted for sex, age, education, body mass index, living alone, anxiety, depression, heart disease, diabetes, and stroke*.

## Discussion

Our study indicated that history of hypertension, duration of hypertension, and high blood pressure were positively associated with dementia among older Chinese people living in an urban community. The MMSE score, which represented the global cognition, was inversely correlated to duration of hypertension, SBP, and DBP. One advantage of the current study was the reliable diagnosis, which was conducted by neurologists with consensus diagnosis at one of the top institutions of neurology in China. Other advantages included the population-based study design with a large sample and the adjustment for confounders, such as sociodemographic characteristics, health behaviors, and medical conditions.

Result from our study was consistent with most of the previous studies which suggested that blood pressure and hypertension are key risk factors for cognitive impairment. In a cross-sectional epidemiological study with 19,836 participants in India/America, an increment of 10 mmHg in DBP was associated with a 7% (95% CI: 1–14%; *P* = 0.0275) higher odds of cognitive impairment based on 6-Item Screener. No independent association was identified between impaired cognitive status and SBP (*OR* = 1.02; 95% CI: 0.99–1.06) or pulse pressure (*OR* = 0.99; 95% CI: 0.95–1.04) (34). However, some studies showed inverse association. A cross-national epidemiological study was conducted in India and America with 4,810 subjects of 55 years and older; in Ballabgarh, India, for every 10 mmHg increase in SBP there was a 10% reduction in cognitive impairment based on a set of cognitive tests (*OR* = 0.90; 95% CI: 0.83–0.97), and there was a 13% reduction in cognitive impairment (*OR* = 0.87; 95% CI: 0.76–0.99) with every 10 mmHg increase in DBP; in the Monongahela Valley, America, a similar association between DBP and cognitive impairment was observed, but it did not remain significant after adjustment for confounders (*OR* = 0.83; 95% CI: 0.65–1.06) (35).

Few studies were reported in the Chinese population. In a cross-sectional study with 1,799 participants (aged 40–85) in a rural area of Xi'an, stratified multivariate analysis revealed a positive relation between SBP (when regarded as continuous data) and cognitive impairment (MMSE) in patients aged 40–49 years (*OR* = 1.35 per 10 mmHg; 95% CI: 1.04–1.75; *P* = 0.025) and 50–59 years (*OR* = 1.19 per 10 mmHg; 95% CI: 1.03–1.37; *P* = 0.019). The analysis turned out to be insignificant for patients aged 60–69 years (*OR* = 0.88 per 10 mmHg; 95% CI: 0.73–1.06; *P* = 0.171) and ≥70 years (*OR* = 0.93 per 10 mmHg; 95% CI: 0.77–1.11; *P* = 0.416). Results similar to those obtained for SBP were obtained for DBP, mean arterial blood pressure, and high blood pressure as well (20). In a prospective observational study with four rural counties in China, 2,000 rural Chinese people aged 65 years and older (median age 70, range 65–92) participated in a baseline evaluation. Two and a half years after baseline the evaluation, a follow-up evaluation of 1,737 subjects was conducted. Cognitive decline based on a set of cognitive tests was derived as the difference between baseline and follow-up scores. Untreated hypertension was associated with greater cognitive decline in this Chinese cohort (19).

The possible mechanism explaining the effect of hypertension on cognitive impairment is not yet clear. A number of autopsy studies (36, 37) have shown that the probability of dementia manifestation for a given level of Alzheimer's disease (AD) pathology is increased by the presence of cerebrovascular pathology, which is strongly linked to hypertension (38–45). Animal studies suggest that cerebral ischemia may be involved in the initiation of AD through upregulation of amyloid precursor protein gene expression (46–48), promotion of amyloid precursor protein cleavage into beta-amyloid peptides (49), or reduction in beta-amyloid peptide clearance (50). Uncontrolled hypertension appears to predict the level of neurofibrillary tangles and neuritic plaques (pathologic indicators of AD) in the brain (51–53), which could be a direct effect of hypertension on AD pathology. Blood pressure may be related to AD initiation or progression through mechanisms that involve beta-amyloid peptides (54), which aggregate to form neuritic plaques.

Some limitations existed in our study. Firstly, we failed to draw conclusions about causal relationship between hypertension and cognitive impairment, especially in the case of the disease that is both age-related and associated with a biased participation rate, from this cross-sectional study design. Secondly, we have adjusted as many potential confounders as possible in the logistic regression model, but we still could not exclude the possible influence of uncollected confounders, such as life experience (e.g., interests, hobbies, leisure activities) and ones' innate intelligence, which could also reflect cognitive reserve. Thirdly, spot blood pressure measurement might not represent the whole situation of blood pressure level. But for each participant, we measured the blood pressure at the same time in the early morning; therefore, it is better than those measured at other time points. Finally, our study site lies in the urban center of Shanghai, and the participants had higher education than most of the others in China. Therefore, the results could not be generalized to the whole Chinese population.

Our results suggest that hypertension and high blood pressure may be potential risk factors for dementia. Blood pressure management for the elderly may be important for maintaining cognitive vitality. The association between blood pressure and risk of cognitive impairment needs to be further studied and validated by prospective studies with longer follow-up time in older population.

## Author contributions

This work was conceptualized by DD, YS, and ZH and all of them approved the protocol. Data collection was done by DD, QZ, QG, XL, and LZ. Statistical analysis was undertaken by XL, WD, and JL. XL, YS, LT, and DD prepared the manuscript. YS and DD are the guarantors of this paper.

### Conflict of interest statement

The authors declare that the research was conducted in the absence of any commercial or financial relationships that could be construed as a potential conflict of interest.
